# Transgenic Cotton (*Gossypium hirsutum* L.) to Combat Weed Vagaries: Utility of an Apical Meristem-Targeted *in planta* Transformation Strategy to Introgress a Modified *CP4-EPSPS* Gene for Glyphosate Tolerance

**DOI:** 10.3389/fpls.2020.00768

**Published:** 2020-07-07

**Authors:** Kesiraju Karthik, Muralimohan Nandiganti, Arulprakash Thangaraj, Shweta Singh, Pragya Mishra, Maniraj Rathinam, Manju Sharma, Nagendra Kumar Singh, Prasanta K. Dash, Rohini Sreevathsa

**Affiliations:** ^1^ICAR-National Institute for Plant Biotechnology, Pusa Campus, New Delhi, India; ^2^Amity Institute of Biotechnology, Amity University Haryana, Gurugram, India

**Keywords:** transgenic cotton, weeds, *CP4-EPSPS*, glyphosate, *N*-phosphonomethyl glycine, herbicide tolerance, *in planta* transformation

## Abstract

Weeds burden plant growth as they compete for space, sunlight, and soil nutrients leading to 25–80% yield losses. Glyphosate [*N*-(phosphonomethyl)glycine] is a widely used broad spectrum non-selective herbicide that controls weeds by inhibiting 5-enolpyruvylshikimate-3-phosphate synthase (EPSPS) enzyme and interfering with the shikimate biosynthesis pathway. Cotton (*Gossypium hirsutum* L.) is one of the most important commercial crops grown worldwide for its fiber. We have developed herbicide tolerant transgenic cotton (cv. P8-6) by introgression of a codon-optimized and modified *EPSPS* gene (*CP4-EPSPS*) possessing an N-terminal chloroplast targeting peptide from *Petunia hybrida*. Because of the recalcitrant nature of cotton, a genotype-independent non-tissue culture-based apical meristem-targeted *in planta* transformation approach was used to develop transformants. Although *in planta* transformation methodologies are advantageous in developing a large number of transgenic plants, effective screening strategies are essential for initial identification of transformants. In the present study, the use of a two-level rigorous screening strategy identified 2.27% of T1 generation plants as tolerant to 800 and 1,500 mg/L of commercially available glyphosate (Roundup). Precise molecular characterization revealed stable integration, expression, and inheritance of *CP4-EPSPS* in advanced generations of the promising transgenic events. Further, superiority of selected transgenic plants in tolerating increasing levels of glyphosate (500–4,000 mg/L) was ascertained through reduced accumulation of shikimate. This report is the first of its kind where cotton transformants tolerating high levels of glyphosate (up to 4,000 mg/L) and accumulating low levels of shikimate have been identified. This study not only reiterated the genotype-independent nature of the transformation strategy but also reiterated the translational utility of the *CP4-EPSPS* gene in management of weeds.

## Introduction

Cotton (*Gossypium hirsutum* L.) is one of the most important commercial crop plants grown worldwide for fiber and seeds. India is the second largest cotton producer contributing to approximately 22% of the world production (Directorate of Cotton Development Government of India, 2017; Mohapatra and Saha, 2019). Weeds are one of the very many factors that negatively challenge productivity of cotton accounting to approximately 50–85% of yield losses (Jain et al., 1981; Frisvold et al., 2009; Ayyadurai and Poonguzhalan, 2011; Awan et al., 2015; Ramachandra et al., 2016). During the early stages of growth, high rate of competition exists between weeds and crop plants for nutrition, space, and amenability, leading to a high percentage of plant mortality in the field (Janaki et al., 2015). Chemical herbicides are an effective way to control weeds, but have raised concerns of environmental pollution. Hence, to overcome these weeds, use of integrated weed management system in the field is an option (Sharma, 2008).

Crop improvement through genetic engineering has given the feasibility to engineer plants to combat herbicides being sprayed and thus help in the safety of the crops without major yield losses (Benbrook, 2016; Bohn and Lovei, 2017). Various genes that confer herbicide tolerance have been identified, validated, and introgressed into a multitude of crops including cotton (Awan et al., 2015; Liang et al., 2017; Zhang et al., 2017).

Glyphosate [*N*-(phosphonomethyl) glycine] is a widely used broad-spectrum non-selective herbicide (Funke et al., 2006; Beckie, 2011; Heap, 2014) that controls weeds by interfering with the shikimate biosynthesis pathway and inhibiting the rate-limiting enzyme 5-enolpyruvylshikimate-3-phosphate synthase (EPSPS; Steinrucken and Amrhein, 1980; Gianessi, 2005; Cao et al., 2012; Gong et al., 2016). The *EPSPS* gene derived from the soil bacterium *Agrobacterium tumefaciens* strain *CP4* is widely known for its tolerance toward glyphosate in an array of transgenic crops (Stalker et al., 1985; Kishore et al., 1986; Padgette et al., 1991; Berry et al., 1992; Padgette et al., 1996; Zhou et al., 2006; Dun et al., 2014; Ma et al., 2016; Fartyal et al., 2018; Yi et al., 2018).

In the present study, we provide a meticulous and in-depth utility of a codon-optimized and modified *CP4-EPSPS* gene in cotton. The gene was assembled with varying GC content for plant codon usage, fused with N-terminal *Petunia hybrida* chloroplast targeting signal peptide (CPT) as the shikimate pathway takes place in chloroplast (Della-Cioppa et al., 1986; Chhapekar et al., 2015). Effectiveness of the gene was further validated in rice for its tolerance to glyphosate (Chhapekar et al., 2015).

Non-tissue culture-based transformation strategies using apical meristem facilitated in the development of stable transgenics in several recalcitrant crop species (Kumar et al., 2011; Keshavareddy et al., 2013; Singh et al., 2018). One such apical meristem-targeted *in planta* transformation approach was exploited in the present study for the development of transformants in cotton (Rohini and Rao, 2002; Keshamma et al., 2008; Karthik and Rohini, 2017). Despite various efforts to introgress herbicide tolerance in cotton by different transformation strategies (Bayley et al., 1992; Lyon et al., 1993; Nida et al., 1996; Rajasekaran et al., 1996b; Keller et al., 1997; Awan et al., 2015; Liang et al., 2017; Zhang et al., 2017), this study is the first of its kind to demonstrate development of stable transgenics with the ability to tolerate high glyphosate using a non-conventional transformation strategy. In this direction, the present study demonstrates the utility of a modified *CP4-EPSPS* gene in transgenic cotton and also reiterates the applicability of the *in planta* transformation approach for cotton improvement.

## Materials and Methods

### *In planta* Transformation of Cotton

**FIGURE 1:**
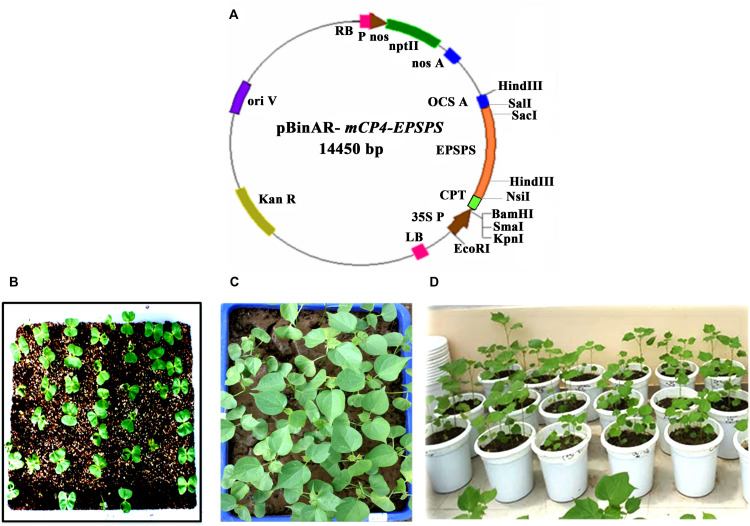
*Agrobacterium tumefaciencs*–mediated *in planta* transformation in cotton. **(A)** Vector map of pBinAR/EPSPS used in the study. Codon-optimized and modified *CP4-EPSPS* gene (1,561 bp) was assembled with varying GC content for plant codon usage and fused at the N-terminal (1–216 bp) with *Petunia hybrida* chloroplast targeting signal peptide (CPT). **(B–D)** Various stages of recovery and establishment of the primary transformants in the greenhouse.

*Agrobacterium tumefaciens* strain EHA 105 harboring the binary vector pBinAR was used for transformation (Figure 1A). The binary vector contained codon optimized and modified *CP4-EPSPS* gene (GenBank accession number is KJ787649) driven by CaMV35s promoter and neomycin phosphotransferase II (*nptII*) gene driven by the nopaline synthase promoter (Figure 1A). For *in planta* transformation, seeds of cotton cultivar P8-6 were surface sterilized and maintained at 32°C for germination. Two-day-old seedlings were used for transformation by following the standardized protocol (Rohini and Rao, 2002; Keshamma et al., 2008; Karthik and Rohini, 2017).

### Glyphosate Tolerance Assays

#### Standardization of Glyphosate Concentration for Screening at Seedling Stage

Seeds of cotton cultivar P8-6 were grown in plastic trays (equal in number) for 8–10 days and at the fourth leaf stage; the plants in each tray were sprayed with glyphosate (200, 400, 600, 800, and 1,000 mg/L) equivalent to 2.5% (vol/vol) Roundup (Monsanto, St. Louis, Missouri, United States). Along with these trays, untreated control was also maintained with plants that did not receive the glyphosate spray. The plants were later scored for their recovery, and the concentration of glyphosate that resulted in the death of wild-type plants was used to select putative transformants.

#### Standardization of Glyphosate Concentration for Screening at Plant Stage

Screening at plant stage was conducted with leaves of 45-day-old wild-type plants using different glyphosate concentrations (50, 100, 150, 200, 250, 300, 500, 1,000, 1,500, 2,000, 2,500, and 3,000 mg/L). Cut leaf discs were placed in Petri plates in three replicates, suspended with 10 mL of glyphosate and maintained for 96 h at room temperature. The leaf discs were later scored for chlorosis.

#### Estimation of Chlorophyll Content in Glyphosate-Treated Leaves

Well-expanded leaves of 45-days-old transgenic and wild-type cotton plants grown under similar conditions were selected for the experiment. Six leaf discs (nearly 200 mg of fresh weight) each of transgenic and wild-type plants were treated with 1,500 mg/L of glyphosate and incubated in Petri plates for 48 h at room temperature in dark. They were later transferred to Eppendorf tubes (Eppendorf, Hamburg, Germany) containing 10 mL of 80% acetone: dimethyl sulfoxide (DMSO) (1:1 vol/vol) solution and incubated in dark for 72 h. Chlorophyll a and b that leached out of the discs were measured spectrophotometrically at 665 and 650 nm, respectively (Hiscox and Israelstam, 1979). Because the assay was conducted with individual T1 generation plants, chlorophyll was estimated in three technical replicates, and the average was presented. The data were analyzed using R Statistical Language version 3.4.3 (2019-2107-30) and Tukey honestly significant difference test to assess significant difference within selected samples.

#### Shikimate Assay

Shikimate assay was performed with leaves (six leaf discs nearly 200 mg of fresh weight for each concentration of glyphosate) of 45-days-old transgenic plants and wild type. Leaf discs were treated with 10 mL of glyphosate of different concentrations (500–4,000 mg/L) for 96 h. Leaf discs of wild-type treated with water were taken as untreated control, and wild-type leaf discs treated with different concentrations of glyphosate were taken as treated control. Further, the leaf discs were collected into an Eppendorf tube; 0.4 mL of 0.25 N HCl was added and incubated for 60 min. It was further ground using a micro pestle, mixed with 0.4 mL 0.25% periodic acid and 0.25% metaperiodate solution for 60 min. After the periodic acid–metaperiodate incubation, a 0.4 mL aliquot of 0.6 M sodium hydroxide with 0.22 M sodium sulfite solution was added (Gaitonde and Gordon, 1958; Koger and Reddy, 2005; Shaner et al., 2005). A shikimate standard curve was developed by adding known amounts of shikimate to vials containing water. Optical density of the solution at 382 nm was determined spectrophotometrically for all the samples taken in the study. The assay was performed with three biological and two technical replicates of each transgenic event. The amount of shikimic acid accumulation in each of the samples was measured, and average values were plotted to evaluate the performance of transgenic events.

### Molecular Analysis of Transgenics for T-DNA Integration

#### Polymerase Chain Reaction Analysis

Genomic DNA from leaves of transgenic and wild-type plants was isolated following a modified cetyl trimethyl ammonium bromide method (Porebski et al., 1997). Polymerase chain reaction (PCR) analysis was performed to amplify a 500-bp T-DNA-specific right border region and 750-bp *nptII* gene using gene specific primers (Table 1). In case of T2 generation plants, PCR analysis was also performed with primers to amplify 420-bp *CP4-EPSPS* gene fragment (Table 1). Polymerase chain reaction amplification was carried out in a thermal cycler (Eppendorf) using the following program: one cycle of initial denaturation at 95°C for 5 min followed by 35 cycles of denaturation at 95°C for 1 min, annealing at 58°C for 1 min, extension at 72°C for 1 min, and one cycle of final extension of 10 min at 72°C. Each PCR reaction (25 μL) consisted of 100 ng of genomic DNA, 2.5 μL of 10× Taq buffer, 10 pM each of forward and reverse primer, 200 μM dNTPs, and 1 U of Taq DNA polymerase (Bangalore Genei, Bengaluru, India) and made up to a final volume of 25 μL with nuclease-free water. While “Blank” had nuclease-free water instead of genomic DNA, negative control contained 100 ng of genomic DNA of wild-type and positive control contained 25 ng of modified *CP4-EPSPS* plasmid.

**TABLE 1:** List of primers used in the study.

Primer ID	Primer sequence (5′–3′)
Right border FP	ATTGGCGGGTAAACCTAAGAG
Right border RP	CTGTATGCGTTGGTGCAATT
*NptII* FP	CCGGAATTCATGATTGAACAA
*NptII* RP	CCCAAGCTTCAGAAGAACTC
*mCP4-EPSPS* FP	GTATCAGAAAGGAGGGCGATAC
*mCP4-EPSPS* RP	CCGAATCCTTGGAGCATCTT
*Ubiquitin* FP	ACACGATCGACAACGTTAAGGCGA
*Ubiquitin* RP	ACGAAGGACAAGATGGAGCGTTGA
*mCP4-EPSPS* FP qRT	GCCAGTGGAGAAACTAGGATTAC
*mCP4-EPSPS* RP qRT	GTATCGCCCTCCTTTCTGATAC

#### Genomic Southern Analysis

For genomic Southern analysis to detect T-DNA copy number, genomic DNA from transgenic and wild-type cotton plants were isolated and purified, and 15 μg of DNA was digested separately with *Hin*dIII (NEB high fidelity, NEB) and *Bam*HI (NEB high fidelity, NEB). The digested DNA samples were then separated by electrophoresis on a 0.8% agarose gel in 1X TAE buffer at constant voltage of 40 V, blotted onto a positively charged nylon membrane (Bio-Rad, Hercules, California, United States) and UV cross-linked. While the membrane with *Hin*dIII-digested genomic DNA samples was probed with DIG PCR-labeled 750-bp *nptII* fragment, the membrane with *Bam*HI-digested genomic DNA samples was probed with DIG PCR-labeled 420-bp *CP4-EPSPS* fragment (Table 1). Hybridization, washing, blocking, and development were carried out according to manufacturer’s instructions (Roche Holding AG, Basel, Switzerland). The membranes were exposed to X-ray film for 1 h in dark and later observed for bands.

#### Western Blot Analysis

Total protein was isolated from 100 mg leaf samples of both transgenic and wild-type plants by finely grinding with liquid nitrogen and later reconstituting in 500 μL of extraction buffer [0.1 M Tris-HCl, 0.5 M EDTA, 30% sucrose, 1% PVPP, 0.1 M KCl, 2% sodium dodecyl sulfate (SDS), 1 mM phenylmethanesulfonyl fluoride, 5% 2-mercaptoethanol; pH 8.8] (Wu et al., 2014). Isolated protein was quantified with Bradford’s assay (Bio-Rad, Hercules, California, United States), and 20 μg of protein was run on SDS–polyacrylamide gel electrophoresis and blotted onto nitrocellulose membrane (Millipore, Burlington, Massachusetts, United States) at a constant voltage (40 V) for 3 h in transfer buffer (1.5 g Tris, 7.2 g glycine and 100 mL methanol made up to 500 mL with distilled water). Initiation of development was performed by blocking the membrane with commercially available NAP blocker (G-Biosciences, St. Louis, Missouri, United States) followed by hybridization of the membrane with protein-specific primary antibodies (Amar Immunodiagnostics, Jubilee Hills, Hyderabad, India) against EPSPS with a dilution of 1:800. Subsequently, the membrane was hybridized with horseradish peroxidase–conjugated secondary antibody (Bangalore Genei) with 1:500 dilution. After blocking with secondary antibodies, the membrane was washed four times with 1X phosphate-buffered saline for 10 min each, followed by the addition of TMB substrate (Promega, Madison, Wisconsin, United States) for color development.

#### Real-Time PCR

Total RNA was isolated from both transgenic and wild-type cotton plants (Spectrum^TM^; Sigma-Aldrich, St. Louis, Missouri, United States); quantity and integrity of total isolated RNA were assessed using a Nanodrop Micro Photometer (Thermo Fisher Scientific, Waltham, Massachusetts, United States). cDNA was synthesized from 2.5 μg total RNA according to manufacturer’s instructions (SuperScript^®^ VILO^TM^; Invitrogen, Carlsbad, California, United States). Gene-specific *CP4-EPSPS* primers and *ubiquitin* primers (internal control) were used to set up the reaction for quantitative PCR (qPCR; Table 1). Polymerase chain reaction program of 5-min initial denaturation at 95°C followed by 40 cycles of denaturation at 95°C for 20 s, annealing at 60°C for 20 s, and extension at 72°C for 15 s was employed. A final melting curve consisting of 95°C for 30 s, 65°C for 30 s, and 95°C for 30 s per cycle was used. Expression analysis was performed with three biological and four technical replicates of each of the events in T2 generation. The Ct values of samples were normalized with the Ct value of ubiquitin (internal control) to calculate Δ Ct value.

## Results

### Development of Transformants in Cotton Harboring the Modified *CP4-EPSPS* Gene

Apical meristem-targeted *in planta* transformation strategy was employed for the development of transgenic cotton. Initially, 180 seedlings were infected with *Agrobacterium*, of which 65 plants recovered. Primary transformants were initially subjected to recovery for 2 weeks under controlled conditions and later transferred to pots in the greenhouse (Figures 1B–D), where they grew normally, flowered, and set seeds. As *in planta* transformation results in the development of chimeric T0 plants, analysis for the selection of putative transformants was carried out in T1 generation.

### Identification of the Suitable Glyphosate Concentration for the Selection of Putative Transformants

**FIGURE 2:**
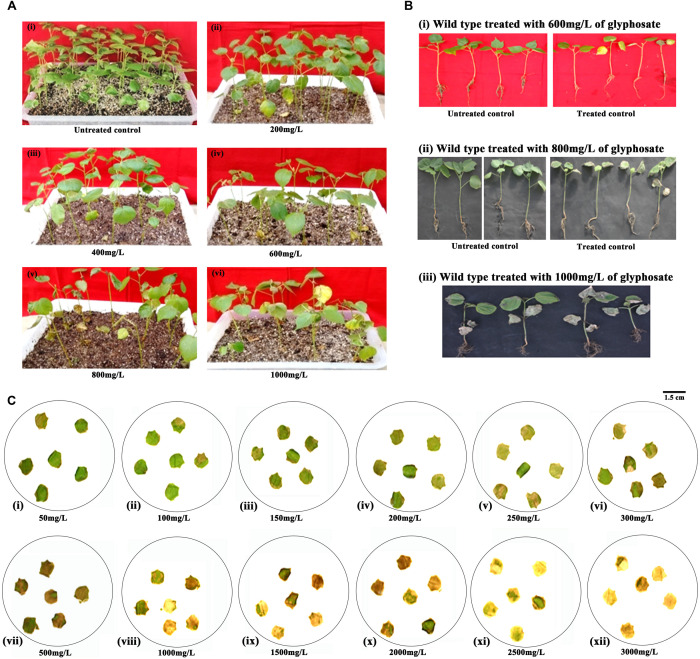
Standardization of glyphosate tolerance assays in cotton cv. P8-6 at seedling and plant level. **(A)** Standardization of glyphosate concentration to be sprayed at seedling level: **(i)** untreated control; seedlings sprayed with different concentrations of glyphosate, **(ii)** 200 mg/L, **(iii)** 400 mg/L, **(iv)** 600 mg/L, **(v)** 800 mg/L, **(vi)** 1,000 mg/L. **(B)** **(i–iii)** Response of the cotton seedlings to different glyphosate concentrations 7–8 days after the spray. **(C)** Standardization of glyphosate concentration in leaf disc assay **(i)** 50 mg/L, **(ii)** 100 mg/L, **(iii)** 150 mg/L, **(iv)** 200 mg/L, **(v)** 250 mg/L, **(vi)** 300 mg/L, **(vii)** 500 mg/L, **(viii)** 1,000 mg/L, **(ix)** 1,500 mg/L, **(x)** 2,000 mg/L, **(xi)** 2,500 mg/L, **(xii)** 3,000 mg/L.

In an effort to identify putative transformants tolerant to glyphosate, the concentration of glyphosate to be used for screening of transgenic seedlings was initially standardized (Figures 2Ai–vi). Approximately 7–8 days after spraying different concentrations of glyphosate, the plants were scored for wilting and chlorosis. Although lower concentrations of glyphosate (200 and 400 mg/L; Figures 2Aii,iii) did not seem to be effective, symptoms were seen in plants that were sprayed with 600 mg/L glyphosate and above (Figures 2Aiv–vi). Higher concentrations of glyphosate (800 and 1,000 mg/L; Figures 2Bi–iii) showed chlorosis and necrosis, eventually leading to death of plants. Based on the response of the wild-type plants, 800 mg/L glyphosate was used for the identification of putative transformants.

Similarly, leaf disc assay with wild-type plants provided insights about the glyphosate tolerance ability at plant level. The assay demonstrated that the leaf discs could not resist glyphosate concentration greater than 150 mg/L (Figures 2Ci–iii). Although symptoms of glyphosate effect was seen with 200 mg/L; 500 mg/L glyphosate and above turned out to be lethal (Figures 2Civ–xii). However, for a stringent plant level selection, 1,500 mg/L was used to identify tolerant transformants.

### Identification and Molecular Characterization of Transgenic Plants in T1 Generation

**FIGURE 3:**
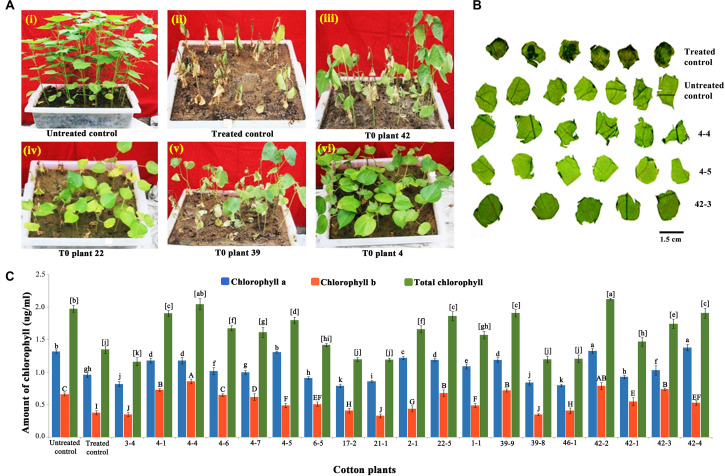
Glyphosate tolerance assays for the identification of putative transformants. **(A)** Seedling level screening: T1 generation and wild-type seedlings were sprayed with 800 mg/L glyphosate **(i)** untreated control, **(ii)** treated control, **(iii–vi)** representative trays containing putative transformants. **(B)** Plant level screening: Response of leaf discs of 45-day-old representative transgenic and wild-type plants to 1,500 mg/L glyphosate. **(C)** Estimation of chlorophyll in the glyphosate treated transgenic plants *vis-à-vis* wild type.

For the identification of putative transformants, three to four leaf stage T1 generation plants were sprayed with 800 mg/L glyphosate. After 7–8 days, as in the treated control (Figure 3Aii; treated control), some T1 generation plants showed necrotic symptoms, turned yellow initially, eventually to brown, and died because of susceptibility toward glyphosate (Figures 3Aiii–vi). However, a set of plants was healthy and on par with the untreated control (Figure 3Ai). Accordingly, a total of 35 plants out of 660 (6.06%) that endured glyphosate spraying were transferred to soil in pots and grown under greenhouse conditions.

Further, to reduce the number of false positives, a second plant level screening was performed with the leaf discs of 35 T1 plants that recovered after glyphosate spraying. Treatment of leaf discs of transgenics and wild-type plants with 1,500 mg/L glyphosate identified 16 tolerant transgenic plants (1-1, 2-1, 3-4, 4-1, 4-4, 4-5, 4-7, 6-5, 17-2, 21-1, 22-5, 39-8, 39-9, 42-1, 42-3, and 46-1) that remained green when compared to other transgenic plants that showed symptoms of bleaching and necrosis (Figure 3B). There was corroboration between the phenotype and the amount of chlorophyll, indicating the superiority of the 16 transgenic plants (Figure 3C). Nearly 2.27% of the putative transformants (16 of 660 plants) were finally selected for molecular characterization on the basis of two stringent glyphosate tolerance assays, as well as normal growth and phenotype.

**FIGURE 4:**
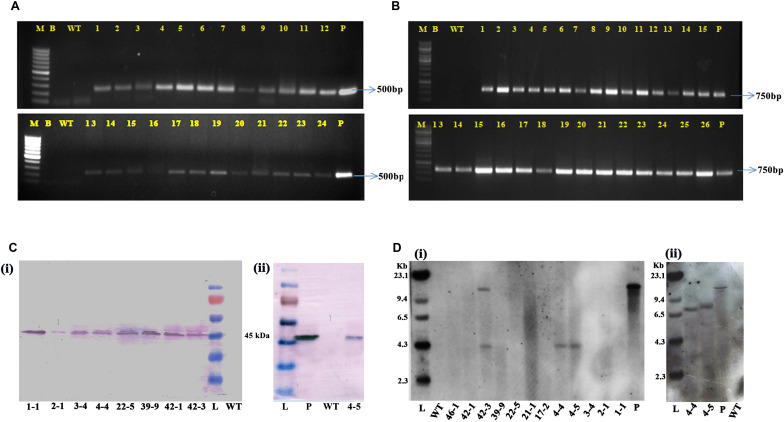
Molecular analysis of transformants in T1 generation. Polymerase chain reaction analysis of T1 generation plants for the amplification of panel **(A)** 500-bp right border region of T-DNA. **(B)** 750-bp *nptII* fragment. Lane M: 1 kb Ladder (Thermo Fisher Scientific); Lane B: Water Blank (all PCR components but without template DNA); Lane WT: wild type; Lanes 1–24 of panel (A) and 1–26 of panel (B): T1 generation transgenic plants; Lane P: positive control (modified *CP4-EPSPS* binary vector). **(C)** Western blot analysis of selected transgenic plants along with wild type. **(i)** Lanes 1–8: transgenic plants (1-1, 2-1, 3-4, 4-4, 22-5, 39-9, 42-1, 42-3). Lane L: Prestained protein ladder; Lane WT: wild type; **(ii)** Lane L: prestained protein ladder; Lane P: purified protein of CP4-EPSPS (30 ng); Lane WT: wild type; Lane 4: transgenic plant 4-5. **(D)** Genomic Southern analysis of T1 generation transgenic plants. **(i)** *Hin*dIII digested genomic DNA probed with DIG-labeled 750-bp *nptII* fragment. Lane L: Lambda DNA *Hin*dIII digest; Lane WT: wild-type DNA; Lanes 3–14: DNA of transgenic plants (46-1, 42-1, 42-3, 39-9, 22-5, 21-1, 17-2, 4-4, 4-5, 3-4, 2-1, 1-1); Lane P: positive control (10 pg DNA of modified *CP4-EPSPS* binary vector). **(ii)** *Bam*HI digested genomic DNA probed with 420-bp *CP4-EPSPS* gene specific fragment. Lane L: Lambda DNA *Hin*dIII digest; Lane 2: 4-4; Lane 3: 4-5; Lane 4: positive control (10-pg modified *CP4-EPSPS* vector DNA); Lane 5: wild-type DNA.

To prove the presence and integration of the transgenes in the selected cotton transformants, PCR analysis performed using primers for T-DNA-specific right border and *nptII* gene-specific primers demonstrated the desired amplicons of size of 500 and 750 bp, respectively (Figures 4A,B). Evidence for the expression of modified *CP4-EPSPS* gene in nine selected transgenic events (1-1, 2-1, 3-4, 4-4, 22-5, 39-9, 42-1, 42-3, and 4-5) was further supported by Western blot analysis (Figures 4Ci,ii). Transgenic plants developed a single intense band of 45 kDa at the expected position as in the positive control (purified CP4-EPSPS), whereas there was no band observed in the wild type.

To assess the integration of T-DNA, 12 T1 generation plants (46-1, 42-1, 42-3, 39-9, 22-5, 21-1, 17-2, 4-4, 4-5, 3-4, 2-1, and 1-1) with robust phenotype that had performed well in both the glyphosate tolerance assays were selected. Genomic Southern analysis confirmed the integration of transgene in three transgenic plants and demonstrated the independent nature of the transgene (Figure 4Di). Two events, 4-4 and 4-5, showed single-copy insertions, whereas plant 42-3 had two copies of the transgene integrated into its genome. Corroboratory results were observed when genomic Southern analysis was performed with the two positive samples (events, 4-4 and 4-5) and probed with 420-bp *CP4-EPSPS* probe (Figure 4Dii), indicating their transformed nature.

### Characterization of Transformants for Inheritance of Transgene in T2 Generation

**FIGURE 5:**
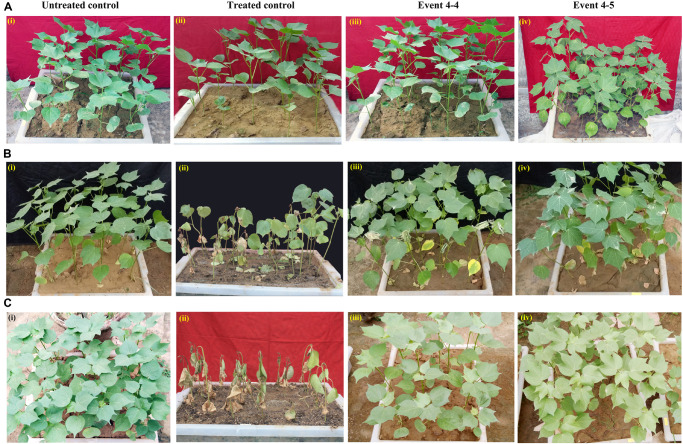
Glyphosate tolerance assay to assess inheritance in T2 generation. **(A)** Wild-type [**(i)** untreated control, **(ii)** treated control] and selected T2 generation transgenic cotton plants [**(iii)** Event 4-4, **(iv)** Event 4-5] maintained under greenhouse conditions. **(B)** Response of transgenic plants *vis-à-vis* wild-type to 1,000 mg/L glyphosate 7–8 days after spray [**(i)** untreated control, **(ii)** treated control, **(iii)** Event 4-4, **(iv)** Event 4-5]. **(C)** Recovery of the transgenic plants *vis-à-vis* wild-type 15 days after glyphosate spray [**(i)** untreated control, **(ii)** treated wild type, **(iii)** Event 4-4, **(iv)** Event 4-5].

To assess the inheritance of the transgenes, analysis of the transgenics in T2 generation was carried out with Southern positive T1 generation events *viz*., 4-4, 4-5, 42-3, and another event 22-5 that performed well in glyphosate tolerance assay. Equal numbers of seeds (approximately 30) of both transgenics and wild-type plants were germinated in individual plastic trays, and fourth leaf stage (approximately 8–10 days after germination) healthy plants (Figures 5Ai–iv) were sprayed with 1,000 mg/L glyphosate. It was observed that after 7 days of spraying, the transgenic plants could apparently withstand the treatment (Figures 5Biii,iv). The treated control plants turned necrotic and eventually died (Figure 5Bii). After 15 days of glyphosate spraying, transgenic events 4-4, 4-5, and 42-3 showed approximately 80% recovery (Figures 5Ciii,iv), whereas line 22-5 had fewer number of plants that has recovered the stress (only three plants), and none recovered among treated wild type (Figure 5Cii). Untreated control plants remained healthy as they were not sprayed with glyphosate (Figure 5Ci). Further proof for the stable inheritance of the T-DNA (Table 2) was provided by segregation analysis carried out with the selected events based on their response to glyphosate spray.

**TABLE 2:** Transgene inheritance analysis in the selected transgenic events.

Selected transgenic events	Total no. of plants tested	No. of plants tolerant to glyphosate	No. of plants susceptible to glyphosate	χ^2^	*p*
Event 4-4	30	24	6	2.06	0.151
Event 4-5	30	23	7	2.022	0.155
Event 42-3	30	20	10	1.88	0.170
Event 22-5	30	3	27	1.1326	0.287

**FIGURE 6:**
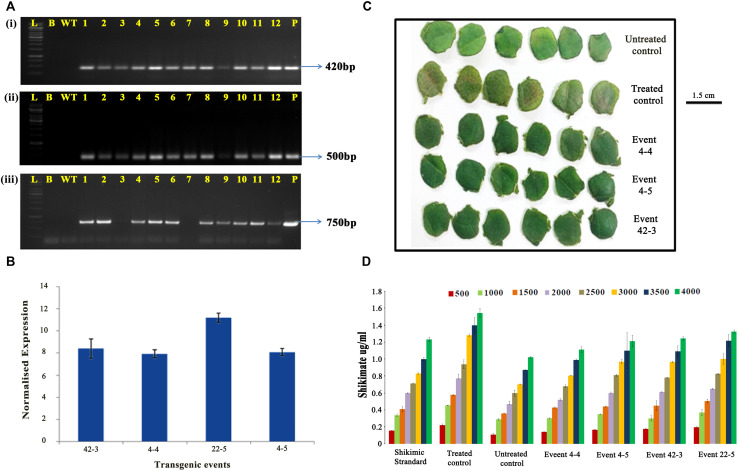
Characterization of cotton transformants in T2 generation. **(A)** Polymerase chain reaction analysis of T_2_ generation plants for the amplification of panel **(i)** 420-bp *CP4-EPSPS* fragment, **(ii)** 500-bp right border region of T-DNA, and **(iii)** 750-bp *nptII* fragment. Lane L: 1 kb ladder (Thermo Fisher Scientific); Lane B: template blank; Lane WT: wild type; Lane 1-12: transgenic plants (three lanes each of events 4-4, 4-5, 42-3, and 22-5); Lane P: positive control (modified *CP4-EPSPS* vector). **(B)** Quantitative RT-PCR analysis of transgenic plants for transcript accumulation of modified *CP4-EPSPS*. **(C,D)** Estimation of shikimate accumulation in the transgenic plants *vis-à-vis* wild type. **(C)** Representative picture showing the response of leaf discs treated with 3,000 mg/L glyphosate taken for shikimate assay. **(D)** Graphical representation of shikimate accumulation in transgenic events *vis-à-vis* wild type incubated with different concentration of glyphosate for 96 h.

The stable inheritance of the transgene was further confirmed by PCR in the plants that survived glyphosate spray using primers specific for *CP4-EPSPS*, T-DNA right border and *nptII* gene (Figures 6Ai–iii). To corroborate integration, expression, and efficacy, the selected transgenic plants, when further assessed for transcript accumulation by qPCR (Figure 6B), showed varied levels of expression of the modified *CP4-EPSPS* gene between 8- and 12-fold.

Quantification of shikimic acid in T2 generation plants *vis-à-vis* wild type showed that the leaf discs of selected transgenic lines seemed to be performing consistently under the imposed stress (Figure 6C). Transgenic event 4-4 had the lowest amount of shikimate accumulation in all glyphosate concentrations (500–4,000 mg/L) in accordance with the shikimate standard. Transgenic events 4-5 and 43-2 showed similarity in the accumulation of shikimate (ranging between 1.0 and 1.2 μg/mL) in leaf samples under all the concentrations of glyphosate (Figure 6D). Although a higher amount of shikimate accumulation was observed in event 22-5, it was considerably lower than that of the treated control, which accumulated maximum amount of shikimate (>1.5 μg/mL). Untreated control had the lowest amount of shikimic acid accumulation in the plant (1.0 μg/mL).

## Discussion

Use of herbicides in modern agriculture has emerged as an integral approach to tackle the problem of weeds as they provide cost-effective increase in agricultural productivity. Because of herbicide usage, there has been at least 12% increase in yield owing to reduced weed competition (Jabran, 2016). Despite the availability of cheap agricultural labor force, large quantities of herbicides are being used in developing countries that could destabilize the functions of soil ecosystem due to high sensitivity to chemicals (Fletcher, 1960).

Glyphosate, one of the widely used herbicides effective in controlling weed populations, is not synthesized in animals as they do not produce aromatic amino acids (Steinrucken and Amrhein, 1980; Gianessi, 2005; Cao et al., 2012; Gong et al., 2016). Hence, glyphosate is less toxic to humans and highly toxic to plants. Resistance to glyphosate has therefore been a pertinent endeavor in crop improvement programs with conscious inputs from breeders and biotechnologists (Peters and Strek, 2018). Resistance to herbicides was among the first traits for which biotechnological approaches were applied (Wilkins et al., 2000). Biotechnological intervention in cotton improvement programs has been long standing with continued inputs toward many traits, including herbicide tolerance using varied genes (Rajasekaran et al., 1996b; Awan et al., 2015; Liang et al., 2017; Zhang et al., 2017). Nevertheless, this study is the first ever comprehensive report demonstrating development, analysis and efficacy of transgenic cotton with a modified *CP4 EPSPS* gene.

Considering the recalcitrant nature of cotton toward tissue culture regeneration, the demonstration of genotype-independent nature of transformation protocols assumes significance (Keshamma et al., 2008). As the initial transformants are chimeric, stringent screening using glyphosate was opted to identify putative transformants in T1 generation. The crux of the study was in the use of a stringent two-level screening of T1 generation plants, at seedling as well as at plant level. This strategy was deliberately designed to select the transformants that could withstand high levels of glyphosate, thus avoiding the advancement of false positives. Although approximately 6% of the seedlings survived the initial glyphosate (1,000 mg/L) challenge, 2.27% of the plants were finally selected for molecular characterization. This success in the identification of transformants in a difficult to transform crop such as cotton is highly commendable (Keshamma et al., 2008).

The foolproof efficacy of transgenics could be concluded by their consistent performance as shown by four events in T2 generation when treated with 1,000 mg/L of glyphosate and their subsequent recovery from the imposed stress. This further reiterates the superiority of the gene, *CP4 EPSPS* in conferring tolerance to high levels of glyphosate (Chhapekar et al., 2015) due to the presence of N-terminal *P. hybrida* CPT. In addition to the molecular characterization to confirm the transgene integration and inheritance, the study also demonstrated the superiority of the selected transformants through the ability to accumulate shikimate. It is known that EPSPS is a rate-limiting biochemical in the shikimate pathway that occurs in the chloroplasts, which is competitively inhibited by glyphosate. Treatment of plants with glyphosate inhibits the EPSPS activity leading to increased accumulation of shikimic acid (Berry et al., 1992). Studies have demonstrated this phenomenon in glyphosate sensitive wild-type cotton plants but reduced shikimate accumulation in transgenic plants (Tian et al., 2010). In the present study, despite high levels of glyphosate (up to 4,000 mg/L), shikimate assay precisely confirmed that the accumulation of shikimate was considerably low in the transgenics as evidenced by their recovery. Three transgenic events were very efficient in regulating shikimate accumulation even at higher concentrations of 4,000 mg/L as depicted by chlorophyll retention. This study is the first of its kind that has shown regulated accumulation of shikimate even at 4,000 mg/L in transgenic events (Rajasekaran et al., 1996a, b; Awan et al., 2015; Liang et al., 2017; Zhang et al., 2017).

The study explored the utility of an Indian cotton cultivar, P8-6, in the development of transgenics by employing an apical meristem-targeted *in planta* transformation strategy. Further, the findings of the study also ascertained the role of the modified *CP4-EPSPS* gene possessing the chloroplast transit peptide (CTP) to target the transgene to chloroplast for tolerating increased glyphosate concentrations in transgenic plants. The transgenics thus developed can form an important pool of resource for introgression of herbicide tolerance into other cotton genotypes and aid in weed management.

## Data Availability Statement

All datasets generated for this study are included in the article/supplementary material.

## Author Contributions

KK has developed the transgenic plants. KK, MN, and AT performed the molecular analyses. KK has written the manuscript. SS, PM, and MR helped KK in preparing the manuscript. NS, PD, and MS have critically edited the manuscript. RS is responsible for acquisition of funds, designing experiments, editing, and revising the manuscript. All the authors have read the manuscript and declare no conflict of interest.

## Conflict of Interest

The authors declare that the research was conducted in the absence of any commercial or financial relationships that could be construed as a potential conflict of interest.
